# Molecular Spectroscopy for the Biochemical Composition Analysis of Patient‐Derived Pancreatic Cancer Organoids

**DOI:** 10.1002/cam4.70457

**Published:** 2024-12-04

**Authors:** Christian Teske, Katja Liedel, Alexander Hirle, Franziska Baenke, Daniel E. Stange, Jürgen Weitz, Grit Preusse, Gerald Steiner

**Affiliations:** ^1^ Department of Visceral, Thoracic and Vascular Surgery University Hospital Carl Gustav Carus, Technische Universität Dresden Dresden Germany; ^2^ National Center for Tumor Diseases (NCT/UCC), German Cancer Research Center (DKFZ), Faculty of Medicine and University Hospital Carl Gustav Carus, Helmholtz‐Zentrum Dresden – Rossendorf (HZDR) Dresden Germany; ^3^ Department of Anesthesia and Intensive Care, Clinical Sensoring and Monitoring, University Hospital and Faculty of Medicine Carl Gustav Carus Technische Universität Dresden Dresden Germany

**Keywords:** FT‐IR, organoids, pancreatic cancer, PDO, spectroscopy

## Abstract

**Background:**

Pancreatic ductal adenocarcinoma (PDAC) continues to pose profound challenges within the field of oncology due to its notorious resistance to existing therapies and constant high mortality rates. The recent emergence of three‐dimensional patient‐derived organoid (PDO) models marks a significant advancement, opening new avenues for exploring cancer biology and assessing therapeutic approaches.

**Aims:**

The aim of this study focuses on the innovative use of Fourier‐transform infrared (FT‐IR) spectroscopy to analyze PDAC organoids, thus illuminating their biochemical intricacies.

**Materials and Methods:**

In this study, PDAC organoids, cultivated from specimens sourced from cancer patients, were subjected to FT‐IR spectroscopic imaging. By examining the spectral data within the critical fingerprint region (950–1800 cm^−1^), and employing principal component analysis (PCA), biochemical disparities were detected and analyzed.

**Results:**

The results revealed distinct spectral profiles corresponding to different sample preparation techniques, which in turn highlighted variations in protein content and structure. PCA revealed a high homogeneity within classes and minimal passage number influence on spectral profiles, with variations in lipid content and protein profiles. Significantly, the biochemical fingerprint of these PDOs closely mirrored that of the original human tissue samples.

**Conclusion:**

This investigation underscores the efficacy of molecular spectroscopy as a non‐invasive method for profound characterization of PDAC organoids, enhancing our comprehension of tumor biochemistry. The capacity for swift and precise biochemical profiling of PDOs via molecular spectroscopy heralds a promising future for this technique in the realms of cancer diagnostics and personalized medicine.

## 1 Introduction

Pancreatic ductal adenocarcinoma (PDAC), characterized by its aggressive nature high mortality rates, remains a formidable challenge in the field of oncology. Recent studies revealed a median overall survival of 40 months after the patients have been resected and treated with adjuvant chemotherapy [1]. Therefore, it continues to be the fourth leading entity of cancer related deaths [2]. Lately, the development of three‐dimensional, patient‐derived organoid (PDO) models has emerged as a promising avenue for studying cancer biology and evaluating potential therapeutic strategies. PDAC organoids have been shown to recapitulate the diverse chemosensitivity in human cancers and a correlation between drug resistance in organoids and humans has been revealed [3, 4, 5]. However, the precise mechanisms underlying the complexity of resistance patterns remain unclear. Decisive progress in the treatment of PDAC promises patient‐ or tumor‐specific therapy with antitumor drugs by understanding the distinct biochemical patterns. PDO, which in simple terms can be seen as mini‐tumors, play a decisive role here. With the tumor organoids, the therapy can be specifically adapted to the individual tumor disease and thus possibly overcome resistance mechanisms. This requires, among other things, a detailed molecular understanding of biomolecular composition. As our understanding of the molecular intricacies governing pancreatic cancer advances, there is a pressing need for innovative and sensitive analytical techniques capable of unraveling the biochemical complexity inherent to this disease.

While cytological and immunological methods usually only reveal a specific molecular target, molecular spectroscopy captures the entire biomolecular profile of cells and tissues. In the landscape of cancer research, methods of molecular spectroscopy, such as infrared (IR) and Raman spectroscopy, have been examined to provide relevant diagnostic information in PDAC [6, 7, 8]. Matrix‐assisted laser desorption/ionization (MALDI) in time‐of‐flight mass spectrometers (TOF) is also a novel technique that has been used to identify biochemical markers for pathological processes, in particular to identify markers of tumor tissue [9]. However, for the MALDI‐TOF technique the sample has to be prepared, and in addition, only large molecules can be analyzed. In contrast, IR and Raman spectra encompass the molecular signature of many molecules, providing a more comprehensive biochemical profile. Furthermore, the sample does not need to be specially treated and can be analyzed under native conditions. IR spectroscopy stands out as a versatile and non‐invasive technique being a pure optical method that holds immense potential to uncover the biochemical signatures underlying tumor biology. The ability of IR spectroscopy to provide detailed information about the chemical composition of biological samples, based on the unique spectral signatures of various biomolecules, positions it at the forefront of contemporary cancer diagnostics and research.

The study presented here is the first to describe the analysis of PDAC PDO using IR spectroscopy. The great potential of this method lies in the rapid label‐ and stain‐free analysis of the biochemical profile of organoids, whereby in particular intracellular and extracellular matrix and stroma is recorded. The combination of state‐of‐the‐art IR spectroscopy techniques and striving PDO models may offer a comprehensive insight into the molecular composition of PDAC and represents a first pivotal step forward in closing the translational gap between conventional cell culture models and the development of targeting therapies.

## 2 Materials and Methods

### 2.1 Human PDAC Organoids

Human PDAC specimens were obtained from patients admitted to the Department of Visceral, Thoracic and Vascular Surgery at the University Hospital Carl Gustav Carus, Technische Universität Dresden, Germany. Informed consent was given prior to surgical procedure; all research methods were performed in accordance with the Declaration of Helsinki. All samples were diagnosed as PDAC according to the World Health Organization criteria by a board‐certified pathologist. Generation of the PDOs was performed according to pre‐existing protocols described elsewhere [10]. The PDOs studied in this analysis were received from an existing biobank. Organoids were cultured in Matrigel matrix (Corning Life Science) and human PDAC organoid medium DMEM/F12+++ supplemented with Wnt3a‐conditioned medium (50% v/v), noggin‐conditioned medium (10% v/v), RSPO1‐conditioned medium (10% v/v), B27 (1x, Invitrogen), nicotinamide (10 mM, Sigma‐Aldrich), gastrin (1 nM, Sigma‐Aldrich), N‐acetyl‐L‐ cysteine (1 mM, Sigma‐Aldrich), primocin (1 mg/mL, Invivo‐ Gen), recombinant murine epidermal growth factor (mEGF, 50 ng/mL, Invitrogen), recombinant human fibroblast growth factor 10 (hFGF10, 100 ng/mL, PeproTech), A‐83‐01 (0.5 μM, Tocris Bioscience), and N2 (1x, Invitrogen).

For this study, four different PDO were analyzed. Original biobanking labels were replaced for a better understanding (DD1385 = sample #1; DD1391 = sample #2; DD1404 = sample #3; NR078 = sample #4). For the influence analysis of multiple passages, DD1391 (sample #5) and DD1404 (sample #6) were re‐analyzed with a gap of more than 10 passages.

The study protocol was approved by the local ethics committee (Technische Universität Dresden, #EK526122019 and #EK397092023). All experiments were performed in accordance with relevant guidelines and regulations.

### 2.2 PDO Preparation

Organoids were harvested and transferred into a falcon. Mechanically dissociation was done with a fire‐polished glass pipette; DPBS was added to a volume of 10 mL. The solution was centrifuged for 5 min with 300×*g*, and the remnant was discarded. The remaining pellet was either resuspended with 250 μL Recovery Solution (Corning) and incubated on ice for 30 min (undigested) or resuspended with 500 μL TrypLE (Thermo Fisher Scientific) for 5 min at 37°C (digested). The resulting solution was filled up with DPBS to 10 mL and centrifuged for 5 min at 30×*g*. The supernatant was discarded, and the pellet was washed twice with NaCl. The remaining pellet was resuspended in 50 μL NaCl per initially used well. 20 μL drops were placed onto a CaF_2_‐slide. Drops were air‐dried, and slides were stored at −80°C.

### 2.3 FT‐IR Spectroscopic Imaging

FT‐IR spectroscopic images were collected in transmission mode using a FT‐IR spectrometer Vertex 70 coupled with an infrared microscope Hyperion 3000 (Bruker Optik GmbH, Ettlingen, Germany) and a MCT focal plane array detector. The imaging detector was a Santa Barbara Focalplane (Goleta, CA, USA) MCT 64 × 64 array detector. The 15‐fold Cassegrainian objective with a numerical aperture of 0.4 imaged a sample area of approx. 175 × 175 μm^2^. From each sample, 2 × 2 individual FT‐IR spectroscopic images with a total dimension of 350 × 350 μm^2^ were captured. Reference spectroscopic images were recorded from the pure calcium fluoride window. For all measurements, a total number of 100 interferograms (scans) were co‐added. The interferograms were Fourier transformed applying Happ‐Genzel apodization and zero filling factor of 1. The resolution of the raw spectra was 6 cm^−1^. Spectra of the sample image were ratioed against the spectra of the reference image and converted to absorbance values.

### 2.4 Data Processing

Evaluation of spectral data was performed using the Matlab Package (Version 8.1, Math Works Inc. Natick, MA, USA). In order to minimize the data volume, only the so‐called fingerprint region between 950 and 1800 cm^−1^ was considered. Data pre‐processing involved a quadratic baseline correction by using the *msbackadj* function of the Statistics Toolbox of Matlab. The baseline correction was performed to reduce influences of light scattering due to the spherical shape of organoids. Spectra with a maximum absorbance larger than 1.5 or with a minimal intensity of the amide I band at 1650 cm^−1^ smaller than 0.02 were empirically identified as outliers and removed from the data set. Finally, the selected spectra were area‐normalized to eradicate spectral differences due to sample thickness or variation in the density of cells and pulp suspensions. In order to compensate variations in the layer thickness and concentration of the organoids, the spectra were area‐normalized for further analysis. Principal component analysis (PCA) was performed on the covariance matrix by using the Matlab function *eig*. Afterwards, score values were reshaped to the sample image.

The *t*‐test for the score values of each PC was performed with the Matlab function *t‐test*. The significance level was set at *α* = 0.01.

Hierarchical cluster analysis of the mean spectra, calculated from the area‐normalized spectra of each sample, was also performed with the Matlab package. The pairwise distance between the samples was calculated with the function *pdist* by using the Euclidean distance measure. The hierarchical cluster tree was generated with the *linkage* function using the weighted average distance and finally displayed by the *dendrogram* routine.

## 3 Results

**FIGURE 1:**
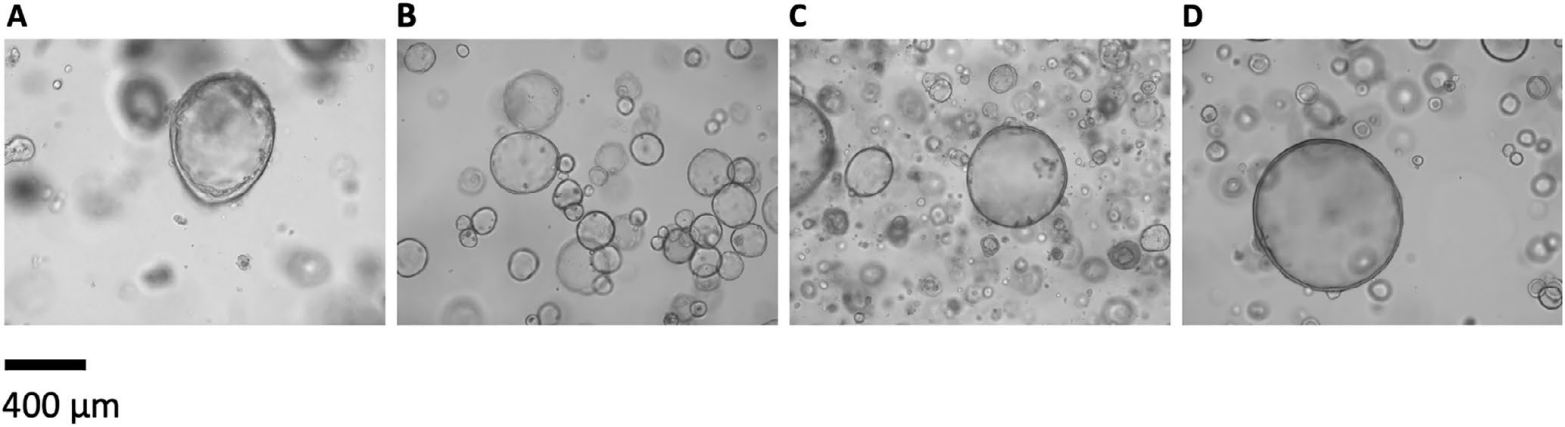
Microscopic images of the analyzed 3D organoid model cultures. (A) sample #1; (B) sample #2; (C) sample #3; (D) sample #4.

Given the complexity of PDO models, four different primary, chemotherapy‐naïve PDAC organoid lines were chosen for IR spectroscopic analysis. Microscopic images from the selected organoids (Figure 1) show the slightly different growth features; however, the bubble‐shape pattern is similar which is in line with recent organoid studies. Mean age of the patients was 69.5 years (3 female, 1 male). One patient died during the hospital stay of the index operation. Mean survival of the other patients was 18 months, dying from recurrent tumor growth or metastasis. More details are found in Table 1.

**TABLE 1:** Details of PDO's associated human pancreatic cancer.

Organoid	Age	Gender	pTNM	CTx‐treatment naïve?
#1	69	Female	pT2, pN1, M1 (lymph)	Yes
#2	60	Male	pT1c, pN1, pM0	Yes
#3	66	Female	pT2, pN2, pM1 (lymph)	Yes
#4	83	female	pT3, pN0, pM0	Yes

**FIGURE 2:**
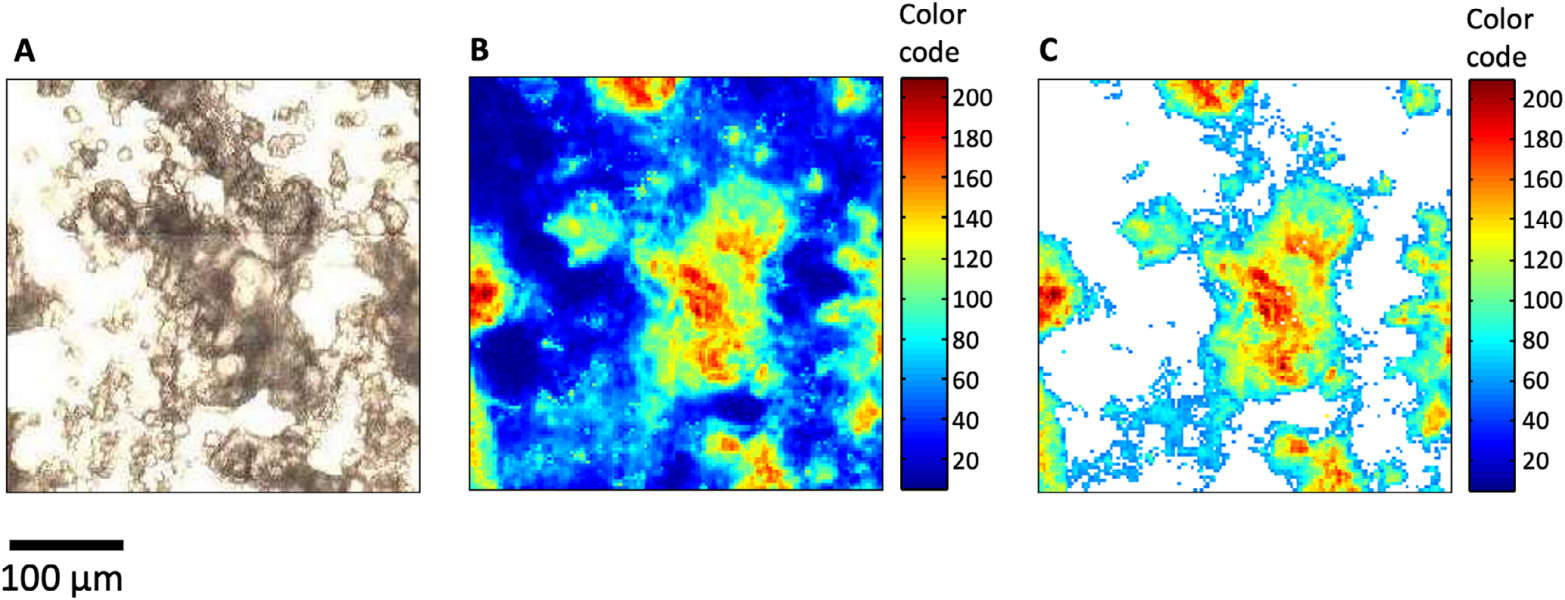
(A) Microscopic white light image of the selected sample area. (B) IR spectroscopic image of the raw data. The contrast is calculated from integral absorbance of the spectral range 950–1800 cm^−1^, converted into a color code. (C) IR spectroscopic image of the integral absorbance after selection of the spectra. Spectra that were removed from the data set are shown in white.

For the spectroscopic investigations, sample areas were selected in which the organoids occur as thinly and evenly distributed as possible. This was done to ensure that predominantly individual organoids are measured in transmission light. Figure 2 shows an example of a recorded sample area (Figure 2A), the corresponding IR spectroscopic image (Figure 2B), and the sample image after applying the spectra selection routine (Figure 2C). White pixels in Figure 2C indicate spectra that have been removed from the data set by applying outlier criteria. The contrast in the spectroscopic image (Figure 2C) corresponds very well with the morphology of the sample (Figure 2A). However, variations in the molecular composition cannot yet be deduced from this.

**FIGURE 3:**
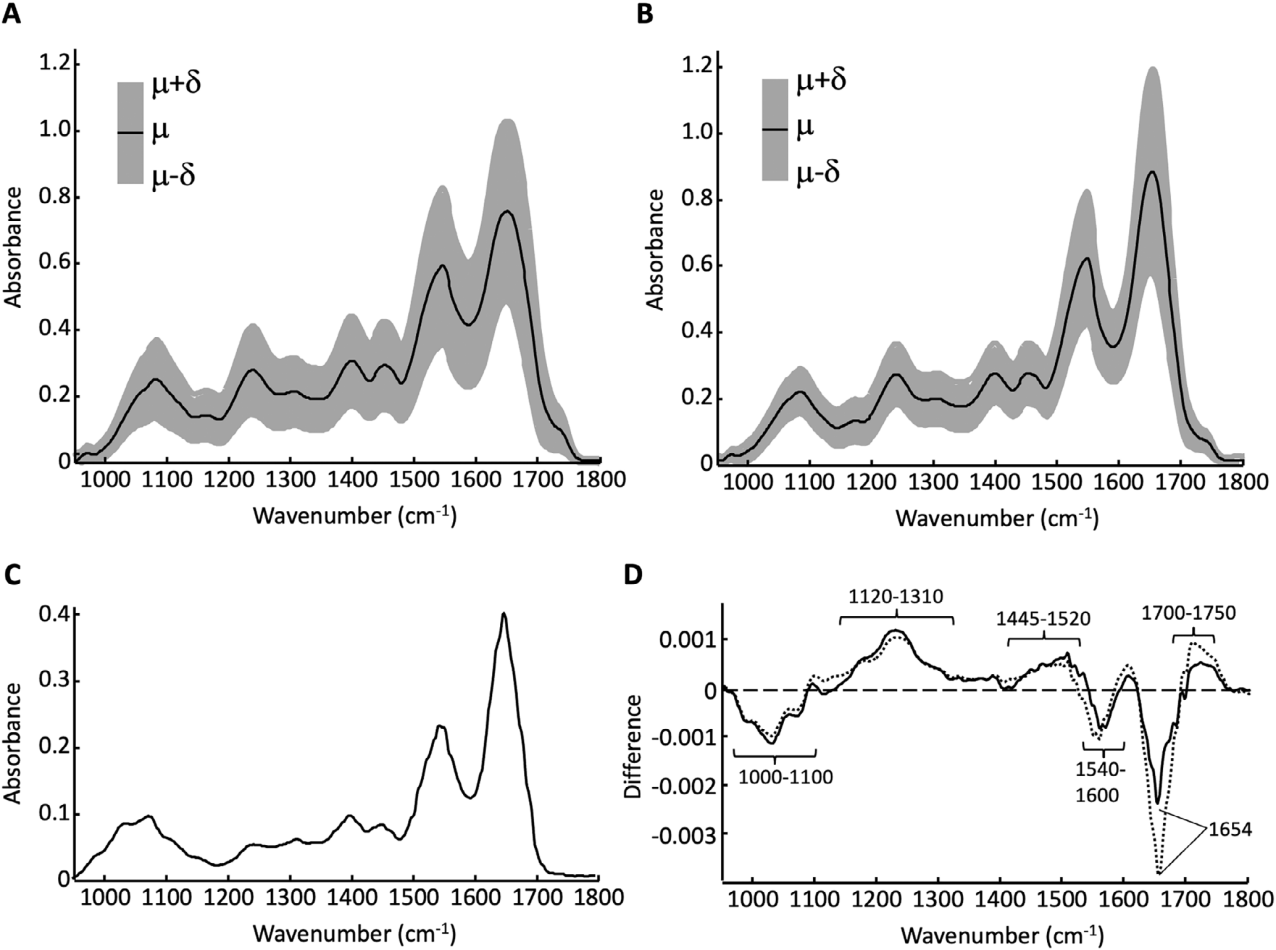
Mean spectrum (μ) and standard deviation (∂) for (A) organoid samples of the undigested class and for (B) organoid samples of the digested class. (C) Mean spectrum of the matrix and (D) difference spectra calculated from the normalized mean value spectra of “undigested”—“matrix” (solid line) and “digested”—“matrix” (dashed line).

For an initial evaluation of the selected spectra, Figure 3 shows the mean spectrum and the standard deviation, calculated from all samples of the classes undigested (Figure 3A) and digested (Figure 3B). Differentiation of these two classes was performed to reveal underlying methodological intricacies in PDO preparation.

For the assignment of the bands and spectral signals, please refer to Table S1. It should also be noted at this point that the exact assignment of bands to molecular structure groups is sometimes very difficult. This applies in particular to the range between approximately 1000 and 1200 cm^−1^. For this reason, reference is only made to the corresponding substance classes and not to specific biochemical substances.

The amide I band between 1600 and 1700 cm^−1^ and the amide II band between 1500 and 1600 cm^−1^ dominate the spectral profile. Other prominent signals occur around 1458 and 1396 cm^−1^ which can be assigned to CH_x_ deformation vibrations. The band around 1230 cm^−1^ is assigned to the amide III vibration. The broad profile between 1000 and 1150 cm^−1^ is mainly composed of vibration modes of phosphate groups and functional groups of carbohydrates. Basically, the spectral profiles of both classes show the typical pattern of IR spectra of cells and tissue. This shows that IR spectra of organoid samples can also be recorded with a high degree of significance for biochemical composition.

Variations in the scattering are noticeable, particularly in the amide I and amide II bands. In general, the spectra of the digested samples (Figure 3B) show a lower standard deviation than the spectra of the undigested samples (Figure 3A). The mean spectrum of the matrix (Figure 3C) is also very similar to the spectral profiles of the plots in Figure 3A,B. This is understandable, as the matrix gel mainly consists of proteins, according to the manufacturer. Carbohydrate components show stronger absorptions, particularly in the range between approximately 1000 cm^−1^ and 1150 cm^−1^. In order to better illustrate the differences, especially with regard to the evaluation of the actual organoid spectra, Figure 3D shows the difference spectra, calculated from the area‐normalized mean spectra of the undigested (Figure 3A) and digested (Figure 3B) PDO samples minus the mean spectrum of the matrix, plotted in Figure 3C. The matrix gel is characterized by a higher protein content, which is particularly evident from the strong negative difference signals between 1540 and 1600 and 1620 and 1700 cm^−1^ with the minimum at 1652 cm^−1^. The broad negative difference signal between approximately 1000 and 1100 cm^−1^ is assigned to absorptions of carbohydrates and their derivatives, among other things. However, it should be noted that a precise assignment is not possible. Both spectra of the organoid classes show a positive difference signal in the range between 1120 and 1310 cm^−1^. In this spectral region, various vibrations of carbonyl groups and their derivatives, as well as polysaccharides, occur. Furthermore, vibrations of phosphate groups can contribute to the stronger absorption of the organoid samples, especially in the region from about 1210 to 1260 cm^−1^.

**FIGURE 4:**
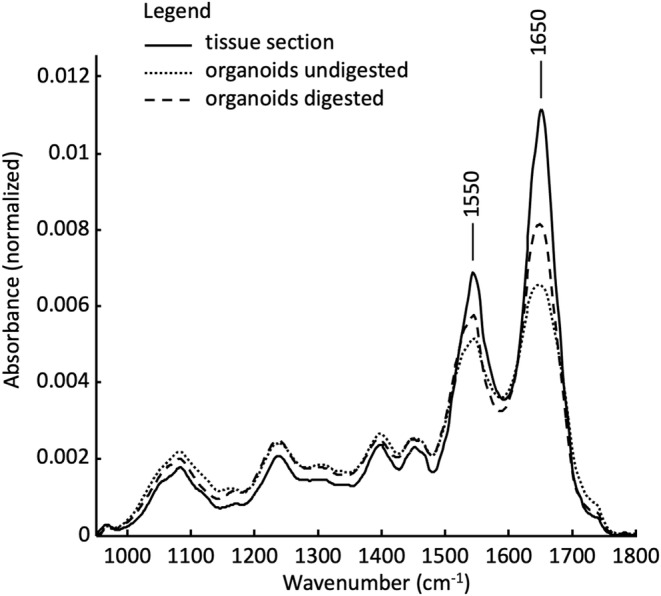
Calculated mean spectra of IR spectra of pancreatic cancer tissue sections, of undigested and digested organoids samples. For better comparability of the spectral profiles, each mean spectrum is area normalized. Mean spectra of pancreatic cancer tissue were adopted [6].

Although there are differences in the mean spectra of undigested and digested organoids, they seem to be very similar to spectra of pancreatic cancer tissue sections. Unstained thin sections of pancreatic tumor tissue have already been studied in detail [6]. In Figure 4 the mean spectra of tissue thin sections, undigested and digested organoid samples are plotted together. The mean spectrum of tissue sections was calculated from all pancreatic tumor samples studied before [6]. The mean spectra of the organoids are identical to the mean spectra in Figure 3. The very high similarity of the spectral profiles is clearly recognizable. Only the amide I and amide II bands at 1500 and 1650 cm^−1^, respectively, show differences mainly in intensity. This comparatively higher protein content of tissue sections is primarily due to the increased expression of collagen in the pancreatic tumor tissue. It can therefore be assumed that the registered spectra of the organoids represent cells of the pancreatic tumor. The similarity of organoid and tissue spectra highlights the translational approach and biochemical complexity of PDO as well as the analytical sensitivity of FT‐IT spectroscopy.

The plots, shown in Figure 3, do not provide any information on how large the deviations are between the samples of the respective classes. Furthermore, it is also not clear whether the passage has an influence on the spectral profile. To investigate this in more detail, the samples of both classes were subjected to a principal component analysis (PCA).

**FIGURE 5:**
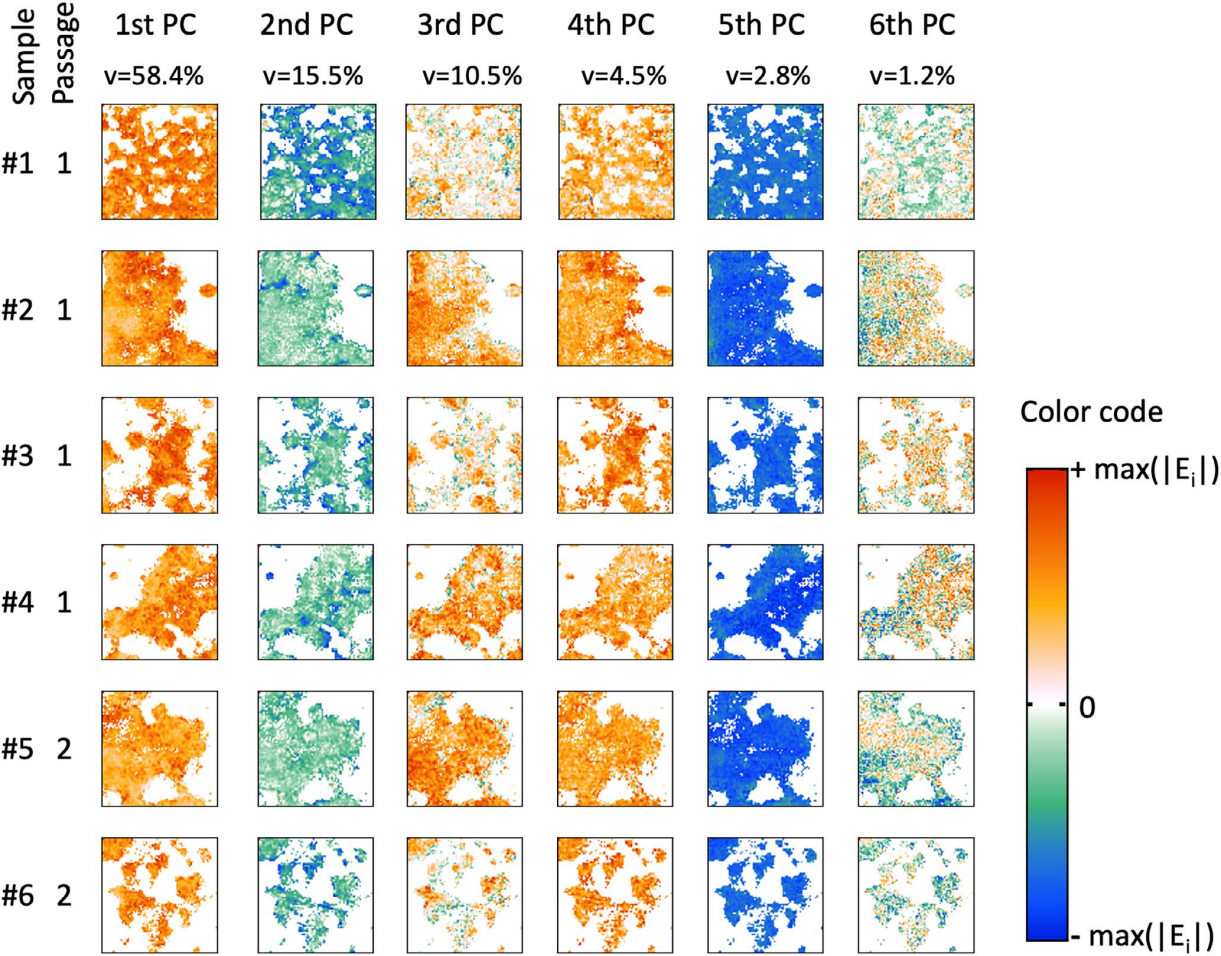
Score images of PCA results of the undigested organoid samples class.

Figure 5 shows the score values of the first six PC of the undigested samples (rows). As can be clearly seen, the score values of the individual samples (columns) differ only minimally. There are also no clear differences between passages (#1–#4 vs. #5 and #6). From this, it can be deduced that there are similar spectral patterns between the individual samples and independent of the passage.

**FIGURE 6:**
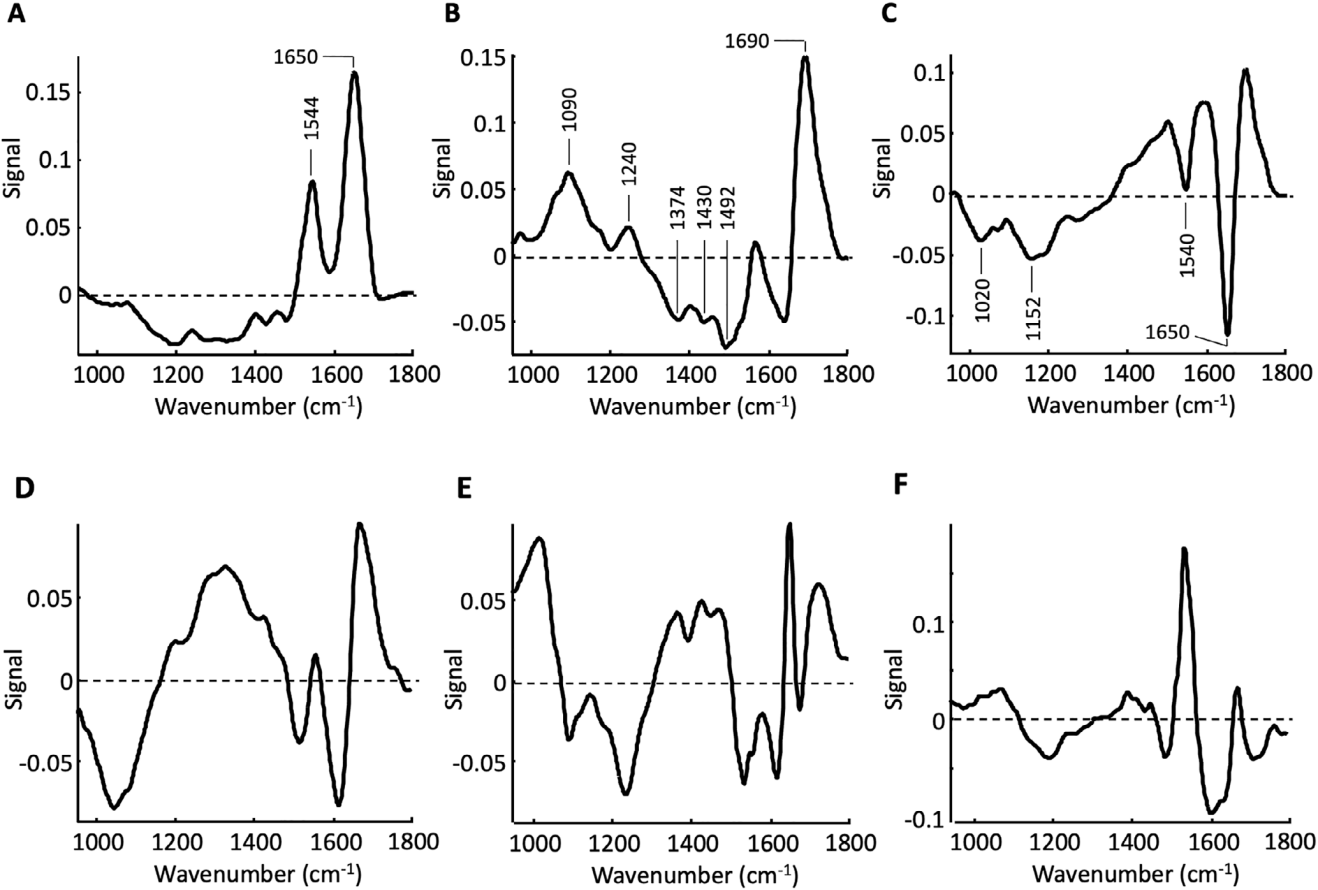
Loading plots of the PCA calculated from undigested organoid samples. (A) First PC, (B) second PC, (C) third PC, (D) fourth PC, (E) fifth PC, and (F) sixth PC.

Differences in the score values between the samples are particularly noticeable for the second and third PCs. Variations in the sixth PC are rather insignificant due to the low variance of 1.2%. The corresponding loading plots have to be considered in order to interpret the differences (Figure 6).

The loading plot of the first PC mainly shows variations in the amide I and amide II bands. The variations within a sample are significantly stronger than between the samples. It is also possible that baseline effects are superimposed in the first PC, which lead to the negative signals and ultimately prevent a more precise assignment of the variations to molecular structures. Nevertheless, in regard to the spectrum of the matrix (Figure 3C) and in particular with regard to the difference spectra (Figure 3D), the first PC can be interpreted predominantly as the influence of the matrix gel, which remained around or between the individual organoids. This is supported by the clear positive signals at 1544 and 1650 cm^−1^ as well as the profile between about 1000 and 1100 cm^−1^ which is probably superimposed by a negative baseline. The loading plot of the second PC shows a strikingly strong positive signal around 1690 cm^−1^, which can be attributed to ν(C=O) stretching vibration modes of the nucleic acids which is also underlined by the position and spectral width up to about 1760 cm^−1^. The positive signals around 1090 and 1240 cm^−1^, which can be attributed to the phosphate groups of the DNA, would also correspond to this. The negative signals localized at 1492, 1430, and 1374 cm^−1^ are associated with CH_x_ vibration modes. Since the score values are consistently negative, this means an increased proportion of CH_x_ groups with a lower proportion of DNA. The second PC can therefore be interpreted as a variation of the lipid content along with the size of the organoids. The loading plot of the third PC, with the two negative signals at 1650 cm^−1^ and 1540 cm^−1^, again indicates slight variations in the protein profile, whereby the comparatively narrow signal at 1650 cm^−1^ can be attributed to the *α*‐helical secondary structure [11]. Further pronounced negative signals are found at 1020 and 1152 cm^−1^, which can be assigned to glycogens. The interpretation of the higher PC's is difficult due to the complex signal patterns and will not be further considered here, especially due to the variations of the score values within the samples being small compared to the second and third PCs.

**FIGURE 7:**
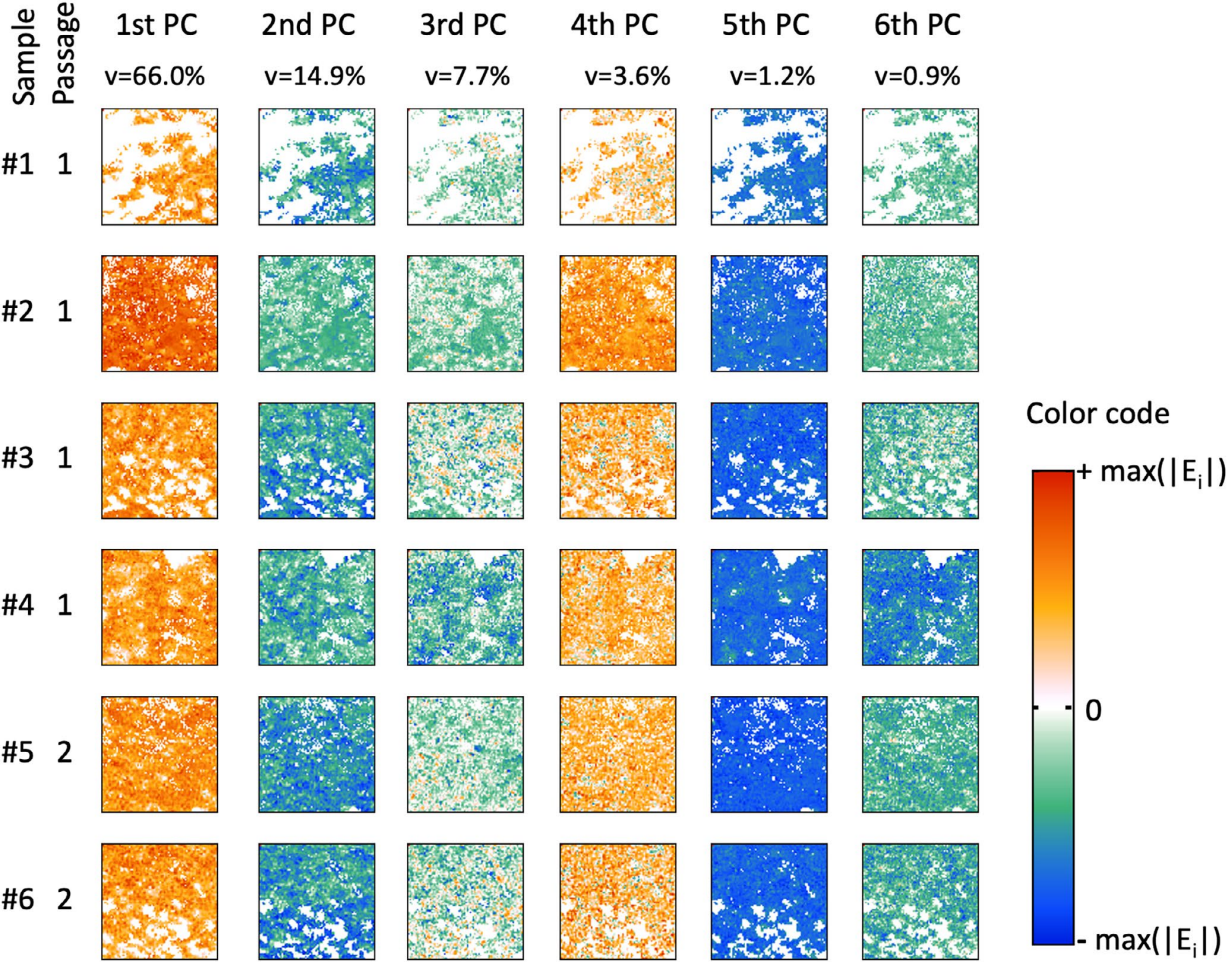
Score images of PCA results of the digested organoid samples class.

In analogy to the class of undigested organoid samples, the digested samples were analyzed and results are presented (Figure 7).

**FIGURE 8:**
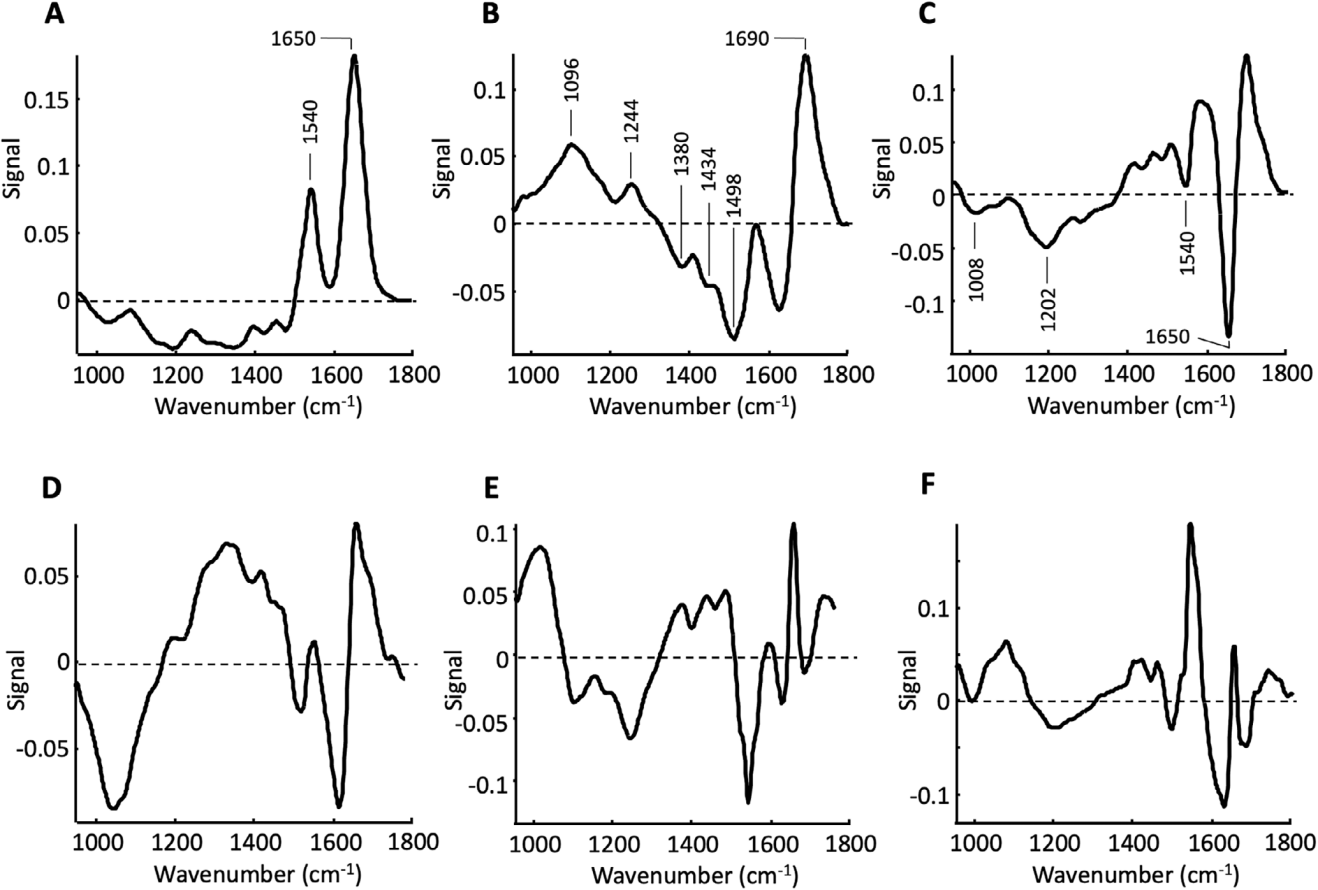
Loading plots of the PCA calculated from digested organoid samples. (A) First PC, (B) second PC, (C) third PC, (D) fourth PC, (E) fifth PC, and (F) sixth PC.

In contrast to the images of the score values of the undigested organoid samples, the digested organoid samples show a greater homogeneity both between and within the individual samples. In analogy to the undigested samples, no clear differences can be found between the passages (#1–#4 vs. #5, #6) in the digested samples either. Similar to Figure 5, variations occur primarily in the second and third PCs. The corresponding loading plots are shown in Figure 8. The signal pattern of all six loading plots is very similar to the signals of the loading plots of the undigested samples (Figure 6). Despite minor deviations in the spectral positions as well as in the signal values, the first three PCs can be interpreted like those of the undigested samples.

To confirm the significance of the score values of each PC in both groups, a *t*‐test was performed. The results of the score values of the first to sixth PC for the undigested and digested samples were calculated and summarized (Table 2). In all cases, the results confirm the hypothesis that the score values of the respective PC originate from a distribution with mean m of the PC. The result of the test with H = 0 means that the hypothesis cannot be rejected at the 1% significance level. The score values of each PC of the undigested and digested samples are therefore significant.

**TABLE 2:** Significance test of score values for (A) undigested and (B) digested samples. H hypothesis of the test; m mean value; c_i_ 1−*α* = 0.9 confidence interval.

A				B			
PC	m	H	c_i_	PC	m	H	c_i_
1.	0.33	0	0.28 to 0.36	1.	0.33	0	0.29 to 0.36
2.	−0.06	0	−0.10 to −0.02	2.	−0.09	0	−0.12 to −0.07
3.	0.03	0	−0.01 to 0.07	3.	−0.02	0	−0.05 to 0.01
4.	0.02	0	−0.02 to 0.06	4.	0.01	0	−0.02 to 0.03
5.	−0.03	0	−0.07 to 0.01	5.	−0.00	0	−0.03 to 0.02
6.	−0.00	0	−0.04 to 0.04	6.	−0.01	0	−0.04 to 0.02

The spectroscopic data, analyzed with PCA, allow some generalizable statements. First, there are no major differences in the molecular profile between the undigested and digested groups. Second, in both groups there are only marginal differences between the passages, that is, the biochemical profile of the organoids remains the same over the observed period. Third, in both groups the protein content varies superficially (see first PC), but these changes are not due to an altered protein composition but rather to variations in the relative protein content. Fourth, in both groups biochemical variations occur within samples mainly assigned to carbohydrates and lipids (see second PC). Fifth, within all organoid samples, small but distinct variations in the secondary structure of proteins can be recognized. This mainly concerns α‐helical substructures. The cause and significance of this finding are still unclear.

**FIGURE 9:**
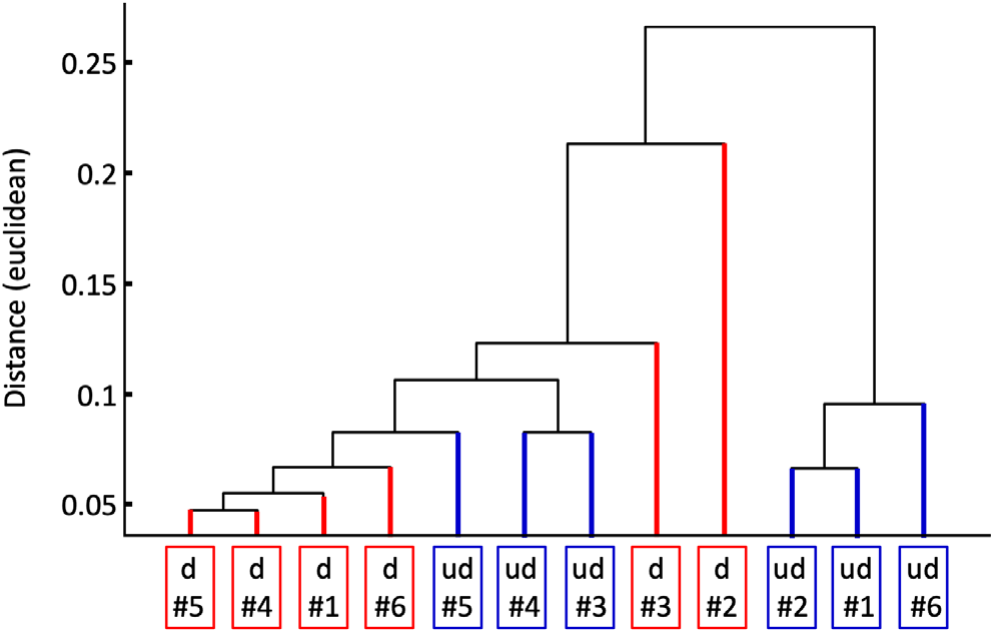
Hierarchical cluster analysis of the mean spectra of undigested (ud, highlighted in blue) and digested (d, highlighted in red) samples. The sample numbers are identical to those in Figures 5 and 7. Mean spectra were calculated from the area‐normalized individual spectra of the respective sample.

While the PCA as method of variance analysis evaluates the differences between the samples within the groups “digested” and “undigested,” a differentiation between the groups can be better examined with the cluster analysis. According to the results of the PCA, the variations between the samples are very similar. The mean spectrum of each sample can therefore also be regarded as representative. The 6 × 2 mean spectra of undigested and digested samples were subjected to a hierarchical cluster analysis (Figure 9).

Although there are differences in the mean spectra between the samples, there is neither a recognizable discrimination between the two groups nor between the passages (see Figures 5 and 7). Hence, the biochemical composition does not differ significantly from the digested or undigested status or from the passage. IR spectroscopy offers a possibility to control or monitor the biochemical state of organoid cultures in a label‐free and objective manner.

## 4 Discussion

This study represents the first research of molecular spectroscopy in the analysis of PDAC organoids. While traditional approaches often focus on genetic markers but fail to address the complete biochemical complexity of the tumors, a deeper molecular understanding is essential to design therapies that are tailored to individual patient profiles and overcome drug resistance effectively. Unlike conventional cytological or immunological techniques that target specific molecules, FT‐IR spectroscopy provides a holistic view of the organoids' molecular composition, aligning closely with the biochemical profiles of native cancer tissues. The results obtained from this novel approach open up new avenues in comprehensively understanding the biochemical landscape of PDAC and may be seen as a first step in assessing PDO models in further patient‐oriented treatment analyses at a molecular level. FT‐IR spectroscopy could enable oncology specialists to design therapies based on the unique molecular landscape of each patient's cancer, moving beyond general chemotherapeutic regimens. By offering insights into protein, lipid, and carbohydrate compositions and their variations across organoid samples, this technique lays the basis for therapies that target specific molecular traits in tumors, significantly advancing personalized treatment options for PDAC patients.

The spectral data from the PDAC organoids, both undigested and digested, reveal significant insights into their biochemical composition and display a strong similarity to the complex patterns of native PDAC tissue [6, 7]. The dominance of amide I and amide II bands is a characteristic feature of cell and tissue IR spectra, underscoring the ability of IR spectroscopy to capture the essential biochemical profile of the samples. Furthermore, analysis of the impact of the digesting method in evaluation of PDO spectra shows only minimal differences mainly caused by the morphologic homogeneity pattern. Therefore, both methods seem to be suitable for further examinations.

The PCA analysis yielded additional layers of information, particularly highlighting the spectral homogeneity within the sample classes and the minimal influence of passage number on the spectral profile. The variations observed in the second and third PCs, predominantly related to lipid content and protein profile variations, underline that IR spectroscopy is sensitive enough to detect subtle biochemical changes within the organoids. This study demonstrates that IR spectroscopy can effectively discern the complex molecular composition of PDAC organoids, which is crucial for understanding tumor biology and the development of targeted therapies. Given the possibility of detecting even mutational alterations in tumor specimens, this method may enhance biomolecular research opportunities in combination with PDO models in the future [12].

The findings of this study can be seen as a research basis for upcoming implications for the field of oncology, particularly in the understanding and treatment of PDAC. By providing a non‐invasive and rapid method to analyze the biochemical profile of cancer organoids, IR spectroscopy may essentially change the way solid tumor entities are characterized and antitumor therapies are evaluated. Moving forward, the paradigm of personalized medical treatment plans and drug assessments may shift from an exclusive focus on individual mutations within surrogate oncogenes to a more comprehensive consideration of holistic biochemical and macromolecular patterns. This may be supported by the potential identification of specific spectral biomarkers or the discrimination of tumor stages using biochemical profile analysis rather than macroscopic clinical evaluation (tumor spread, metastasis). Furthermore, the ability to track organoid responses to environmental stressors through changes in the spectral profile offers a promising tool for the development of decisive, patient‐oriented plans. This aligns with the growing trend toward personalized medicine, where treatments are tailored to the individual characteristics of a patient's tumor [13].

However, there are some limitations to this study in interpretation of the presented results. A relatively small number of PDO samples were analyzed, all derived from chemotherapy‐naïve PDAC patients. The limited sample diversity may restrict the generalizability of the findings, particularly in terms of representing the complex range of biochemical and molecular variability in PDAC tumors across different stages and treatment histories. Although FT‐IR spectroscopy provides detailed biochemical profiles, the clear assignment of spectral bands to specific molecular structures can be challenging, especially in overlapping spectral regions. This may limit the resolution of certain molecular distinctions crucial for refining personalized treatment strategies. Furthermore, the study does not correlate the obtained spectral profiles with actual therapeutic patients' outcomes due to the little number of samples. Future studies need to validate if the spectral patterns observed in PDO correlate with clinically significant treatment responses.

## 5 Conclusion

In conclusion, this study marks a significant step forward in PDAC research. The use of IR spectroscopy to analyze PDAC organoids not only enhances our understanding of the molecular intricacies of this disease but also paves the way for more effective, personalized treatment strategies. As we advance our technological and methodological approaches, the potential to improve patient outcomes in PDAC becomes increasingly attainable.

## Author Contributions

**Christian Teske:** conceptualization (lead), data curation (equal), formal analysis (supporting), funding acquisition (lead), investigation (lead), methodology (equal), project administration (lead), resources (lead), supervision (lead), visualization (equal), writing – original draft (lead), writing – review and editing (lead). **Katja Liedel:** conceptualization (equal), formal analysis (supporting), funding acquisition (supporting), investigation (lead), methodology (equal), software (equal), writing – original draft (supporting). **Alexander Hirle:** formal analysis (equal), investigation (supporting), methodology (supporting), software (equal), writing – original draft (equal). **Franziska Baenke:** conceptualization (equal), data curation (equal), formal analysis (supporting), investigation (equal), project administration (equal), resources (equal), supervision (equal), writing – review and editing (equal). **Daniel E. Stange:** conceptualization (equal), data curation (equal), funding acquisition (equal), methodology (equal), project administration (equal), resources (equal), supervision (supporting), writing – review and editing (supporting). **Jürgen Weitz:** conceptualization (equal), funding acquisition (equal), investigation (equal), project administration (supporting), resources (equal), supervision (supporting), writing – review and editing (equal). **Grit Preusse:** data curation (equal), formal analysis (supporting), investigation (equal), methodology (equal), resources (equal), supervision (equal), writing – original draft (equal). **Gerald Steiner:** conceptualization (equal), data curation (lead), formal analysis (lead), funding acquisition (supporting), investigation (supporting), methodology (equal), project administration (supporting), resources (equal), software (equal), supervision (equal), visualization (lead), writing – original draft (equal), writing – review and editing (equal).

## Ethics Statement

The study was approved by the local ethics committee (Technische Universität Dresden, #EK526122019 and #EK397092023). The patients gave their written informed consent for the research project prior to surgery.

## Conflicts of Interest

The authors declare no conflicts of interest.

## Précis

Molecular spectroscopy is an efficient, non‐invasive method to deeply characterize cancer organoids. The similar biochemical profile compared to native cancer tissue offers a profound swift in cancer diagnostics and personalized medicine.

## Supporting information


**Table S1.** Assignment of IR absorption spectral bands in the region from 950 to 1800 cm^−1^ and their molecular assignments^16^. δ, deformation; ν, stretching; s, symmetric; adopted [1, 2].

## Data Availability

The presented data and the organoid material are available upon reasonable request.
