# Supplementary material for: Molecular Spectroscopy for the Biochemical Composition Analysis of Patient‐Derived Pancreatic Cancer Organoids
Source: Cancer Med. 2024 Dec 4;13(23):e70457. doi: 10.1002/cam4.70457 (PMC11617587; doi:10.1002/cam4.70457)
Supplement: Supplementary file 1 — Table S1. Assignment of IR absorption spectral bands in the region from 950 to 1800 cm−1 and their molecular assignments16. δ, deformation; ν, stretching; s, symmetric; adopted [1, 2]. [file CAM4-13-e70457-s001.docx]

**Molecular spectroscopy for the biochemical composition analysis of patient-derived pancreatic cancer organoids**

Christian Teske, MD^1,2*^; Katja Liedel, M.Sc.^1,2^; Alexander Hirle^1,2^; Franziska Baenke, PhD^1,2^; Daniel E. Stange, MD, PhD^1,2^; Jürgen Weitz, MD^1,2^; Grit Preusse, PhD^3^; Gerald Steiner, PhD^3^

^1^ Department of Visceral, Thoracic and Vascular Surgery, University Hospital Carl Gustav Carus, Technische Universität Dresden, Germany

^2^ National Center for Tumor Diseases (NCT/UCC), Dresden, Germany: German Cancer Research Center (DKFZ), Heidelberg, Germany; Faculty of Medicine and University Hospital Carl Gustav Carus, Technische Universität Dresden, Dresden, Germany; Helmholtz-Zentrum Dresden - Rossendorf (HZDR), Dresden, Germany

^3^ Department of Anesthesia and Intensive Care, Clinical Sensoring and Monitoring, University Hospital and Faculty of Medicine Carl Gustav Carus, Technische Universität Dresden, Germany

**Supplementary Material**

| **Spectral position (cm^-1^)** | **Assignment** |
| --- | --- |
| 1020 | ν(C-O), δ(C-O) |
| 1038 | ν(C-C) skeletal, ν(CH_2_OH), ν(C-O), δ(C-O) |
| 1047 | ν(C-O) of carbohydrates, glycogen |
| 1082 | ν_s_(PO_2_‾) of DNA and RNA |
| 1088 | ν_s_(PO_2_‾) of DNA and RNA |
| 1120 | ν_s_(P-O-C), ν(C-O), carbohydrates |
| 1152 | ν(C-O) of carbohydrates, δ(CH_3_) |
| 1173 | ν(C-O) |
| 1206 | Amide III components, ν(PO_2_‾), |
| 1212 | ν(PO_2_‾) |
| 1240 | Amide III components, ν(PO_2_‾) |
| 1310 | Amide III components |
| 1374 | ν(C-N), δ(C-H), δ(N-H) |
| 1396 | δ(CH_3_), δ(CH_2_) |
| 1408 | δ(CH_3_) |
| 1430 | δ(CH_2_) |
| 1458 | δ(CH_3_) |
| 1492 | δ(C-H), in plane |
| 1500 - 1600 | Amide II |
| 1600 - 1700 | Amide I |
| 1690 | unassigned |

**Supplementary table 1** Assignment of IR absorption spectral bands in the region from 950 cm^-1^ to 1800 cm^-1^ and their molecular assignments^16^. δ - deformation, ν - stretching, s – symmetric; adopted^1,2^.

**References**

1. Infrared and Raman Characteristic Group Frequencies: Tables and Charts, 3rd Edition | Wiley. *Wiley.com* https://www.wiley.com/en-us/Infrared+and+Raman+Characteristic+Group+Frequencies%3A+Tables+and+Charts%2C+3rd+Edition-p-9780470093078.

2. Movasaghi, Z., Rehman, S. & Ur Rehman, Dr. I. Fourier Transform Infrared (FTIR) Spectroscopy of Biological Tissues. *Appl. Spectrosc. Rev.* **43**, 134–179 (2008).
